# The characterization of CellROX™ probes could be a crucial factor in ram sperm quality assessment

**DOI:** 10.3389/fvets.2024.1342808

**Published:** 2024-02-27

**Authors:** Cristina Palacin-Martinez, Luis Anel-Lopez, Mercedes Alvarez, Marta Neila-Montero, Rafael Montes-Garrido, Cristina Soriano-Úbeda, Paulino de Paz, Luis Anel, Marta F. Riesco

**Affiliations:** ^1^Investigación en Técnicas de Reproducción Asistida (Itra-ULE), Instituto de Desarrollo Ganadero y Sanidad Animal (INDEGSAL), University of León, León, Spain; ^2^Animal Reproduction and Obstetrics, Department of Veterinary Medicine, Surgery and Anatomy, University of León, León, Spain; ^3^Anatomy, Department of Veterinary Medicine, Surgery and Anatomy, University of León, León, Spain; ^4^Celular Biology, Department of Molecular Biology, University of León, León, Spain

**Keywords:** CellROX probes, lipid peroxidation, ovine, oxidative stress, reactive oxygen species, sperm

## Abstract

Several authors have demonstrated that low levels of reactive oxygen species (ROS) are necessary for the physiological functions of sperm, such as capacitation, hyperactivation, acrosomal reaction and fertilization. However, high levels of ROS are associated with oxidative stress and detrimental effects on fertility. Consequently, deep characterization of ROS presence using different fluorescent probes could be crucial. In this sense, the study of intracellular ROS localization and the relationships between ROS and other conventional parameters could improve the characterization of sperm quality for semen preservation protocols in rams. In this work, a multiparametric study was carried out by analyzing four experimental groups of ram sperm with different initial qualities: fresh semen (from both breeding and nonbreeding seasons), frozen-thawed semen and, a positive control group treated with hydrogen peroxide (300 μM) as a marker of extreme damage. Sperm analyses, including viability, apoptosis, lipid peroxidation, motility and kinetic parameters, were applied to compare several experimental groups with different sperm qualities. After that, the signals from two different ROS probes: CellROX™ Deep Red (CRDR) and Green (CRG), were examined by flow cytometry (percentage of cells that express ROS) and fluorescence microscopy (intracellular ROS location). Comparing conventional parameters, fresh samples from the breeding season showed the highest sperm quality, while the positive control samples showed the worst sperm quality. Concerning the ROS probes, the CRDR levels were higher in fresh samples from the breeding season than in the positive control and cryopreserved samples. Surprisingly, CRG presented its highest level (P < 0.05) in the positive control group treated with peroxide by flow cytometry. CRDR and CRG presented opposite labeling patterns that were corroborated by fluorescence microscopy, which determined that the probes localized in different parts of sperm. CRDR was found in the sperm mitochondrial region, while CRG was observed in the cell nucleus, suggesting that ROS localization is an important factor. Finally, our study indicates that CRDR is correlated with proper viability and sperm motility, and could be associated with high mitochondrial activity, while CRG is associated with sperm damage.

## 1 Introduction

Oxidative stress is one of the leading causes of male infertility that is a consequence of an imbalance in reactive oxygen species (ROS) (1, 2). This effect has been previously demonstrated by different authors in some mammalian species, including rams (3–5), stallions (6), red deer (7) and bulls (8). Sperm cells are very susceptible to oxidative stress due to the high number of unsaturated lipids that compose their cell membranes (9); for this reason, lipid peroxidation is highly correlated with ROS content in sperm cells (5). Sperm preservation methods (cooling and cryopreservation) are the main cause of oxidative stress in domestic animals (10). Sperm cells suffer detrimental effects due to the drastic temperature differences, including the overproduction of ROS, reduced acrosome integrity or impairment of mitochondrial membrane potential (10). An imbalance in the redox system due to an increase in ROS levels triggers a decrease in sperm quality. Specifically, high ROS levels cause lipid peroxidation, DNA fragmentation, apoptosis, and consequently low fertility (11, 12). ROS include superoxide anions (O2), hydrogen peroxide (H_2_O_2_), proxyl (·ROO), and hydroxyl (·OH) radicals (5). Balanced ROS generation is essential for sperm capacitation, hyperactivation, the acrosomal reaction and fertilization (13–15). Moreover, seminal plasma contains different antioxidants that are very relevant for sperm protection, including enzymatic antioxidants [superoxide dismutase (SOD), glutathione peroxidase (GPX), and catalase (CAT)] and nonenzymatic antioxidants such as vitamins (A, C, and E) (2, 11). High levels of ROS can be compensated for the seminal plasma antioxidant system (11). Nevertheless, an imbalance caused by excessive ROS production and decreased levels of antioxidant enzymes provokes oxidative stress (13, 14).

Therefore, a key point for improving sperm quality analyses should focus on the redox status of sperm (11, 16). This status can be assessed by direct measurements (ROS production) or indirect measurements (malondialdehyde (MDA), high DNA fragmentation index (hDFI), and SOD and GPX levels) (11, 17). In recent decades, the amount of MDA present in sperm cells has been used as a measure of lipid peroxidation (11, 18) and is an indirect measure of ROS production and oxidative damage (18). The reaction of MDA with thiobarbituric acid (TBA) forms a product that can be identified colorimetrically or fluorometrically, and its signal is proportional to the amount of MDA present. Moreover, several authors have noted an association between a high DNA fragmentation index and oxidative stress (19). Different techniques have been used to evaluate DNA fragmentation, in particular Sperm Chromatin Structure Assay (SCSA^®^) technique described by Everson (20) which allows the use of flow cytometry. In addition, there are other techniques used for the evaluation of DNA fragmentation including Terminal deoxynucleotidyl transferase-mediated dUTP-biotin nick end labeling (TUNEL assay), *in situ* nick translation assay (ISNT), Sperm Chromatin Dispersion (SCD) or Single-cell gel electrophoresis assay (Comet) (21).

In recent years, new approaches based on multiparametric analyses by flow cytometry have been developed as innovative technologies to evaluate ROS production in ram sperm (22). Flow cytometry is a useful tool for the evaluation of sperm quality. This technique allows the analysis of a large number of cells, capturing many features of each of them in a few seconds (23). Its ability to analyze multiple sperm characteristics allows for a better understanding of sperm functionality (24), which will enable the development of sperm quality analysis (25, 26). Multiparametric analysis by flow cytometry has been developed to ensure adequate sperm quality to perform fertility studies in different mammalian species, such as humans (27, 28), bulls (29), and stallions (30). Some oxidative stress measurement techniques have been developed for the evaluation of specific ROS and other oxidative species can be carried out by reagents that accumulate intracellularly and become fluorescent upon oxidation (23). These fluorochromes include MitoSOX Red probe used to identify superoxide anion and hydrogen peroxide in human sperm (31), 2′, 7′-dichlorodihydrofluorescein diacetate (H2DCF-DA) to detect hydrogen peroxide (32, 33) or 4,5-diaminofluorescein diacetate (DAF-FM) used for identify nitric oxide in stallion sperm (34).

Recently, novel CellROX fluorescent probes in different colors have been introduced as ROS markers: CellROX™ Deep Red, CellROX™ Orange and CellROX™ Green. CellROX™ Deep Red and Green can detect hydroxyl radicals and superoxide anions in sperm cells (35–37). On the other hand, CellROX™ Orange detects hydroxyl peroxide, hydroxyl radicals, nitric oxide, peroxide nitrile anions and superoxide anions in sperm cells (37, 38). Additionally, CellROX probes can be used in fluorescence microscopy and spectrophotometry, unlike previous probes (35). Therefore, these innovative probes allow for deep characterization and identification of intracellular ROS localization. In the case of sperm cells, this characterization is more interesting because these cells are composed of a head (where the nucleus is located), middle piece (where the mitochondria are placed), and tail (39). ROS can be localized to the mitochondria but also in the nucleus, where they promote DNA damage (40). For this reason, these novel fluorescent probes should be analyzed.

The objective of this work was to validate the efficiency of the fluorescent probes CellROX™ Deep Red (CRDR) and CellROX™ Green (CRG) in identifying the presence of ROS [the hydroxyl radical (OH) and superoxide anion (O_2_)], including their intracellular location and concentrations in ram sperm. In addition, the relationships between the fluorescent probes and different parameters of sperm quality were studied. For this purpose, motility and kinetic parameters, multiparametric flow cytometry analyses (viability and apoptosis), hDFI and, MDA measurements were correlated with the novel fluorescent probes. This evaluation could improve semen preservation protocols in rams.

## 2 Materials and methods

### 2.1 Animal care and sperm collection

The study was performed following the Guidelines of the European Union Council (86/609/EU), modified by 2010/63/UE, according to the national laws (RD 2013) for laboratory animals. The experimental instructions were approved by Animal Care and Use Committee of the University of León (Spain) (ÉTICA-ULE-050-2022). Seven healthy adult Assaf rams were used in this experiment during the breeding and nonbreeding seasons. Rams were owned by the National Association of Assaf Sheep Breeders (ASSAF.E) and kept under uniform nutritional conditions at the Animal Selection and Reproduction Center of Junta de Castilla y León (CENSYRA) located in Villaquilambre (León, Spain). Trained males were used to carry out the experiments (twice weekly semen collection). Fourteen ejaculates (two per male) were collected by an artificial vagina (40°C) in two different seasons: (i) breeding season (BS) and (ii) nonbreeding season (NBS). Immediately after collection, the ejaculates were kept in a water bath at 30°C during the initial evaluation of semen quality. This assessment consisted of volume measurements (graduated tubes) and mass motility (a 5 μL drop on a microscope was prepared with a warmed plate, and a subject score from 0 to 5 was determined).

### 2.2 Experimental groups and sample treatments

Experimental groups with different sperm qualities were used: fresh samples [breeding season (BS) and nonbreeding season (NBS)], frozen-thawed NBS samples and positive control samples from the BS (oxidative stress induction with hydrogen peroxide). The first and second ejaculates from each male were mixed, and each pool was split into two subsamples to obtain the four experimental groups: BS and positive control (PC) from the breeding season and NBS and cryopreserved from the nonbreeding season (Supplementary Figure 1). Samples from the breeding and nonbreeding seasons were diluted to the same volume (1:1) in a dilution medium design by our group -Itra-ULE- (41, 42). This medium was composed by TES solution (325 mOsm/kg) and TRIS solution (325 mOsm/kg), mix to pH= 7.2, D-Fructose solution (325 mOsm/kg), supplemented with 20% egg yolk, penicillin G (sodium salt) (500,000 IU/L), and dihydrostreptomycin sulfate (625 mg/L) (41, 42). After that, the samples were immediately transported to the laboratory in a water bath at 30°C. Once there, the sperm concentrations were determined by a cell counter (Nucleocounter SP-100, ChemoMetec, Allerod, Denmark). Samples were diluted to 1,600 × 10^6^ spermatozoa/mL in TTFM. Immediately, the semen was refrigerated by cooling at a rate of −0.5°C/min from 30°C to 15°C in a programmable bath (CC-K8, Huber, Germany). Positive control samples were submitted to oxidative stress induction by treatment with 3% hydrogen peroxide (Viviar, Valencia, Spain) diluted in PBS for a final hydrogen peroxide concentration of 300 μM for 24 h at 37°C, as described by Soliman et al. (16). Cryopreserved samples were diluted to the same volume (1:1) in TTFM supplemented with 20% clarified egg yolk and 4% glycerol made by our group (41). Samples were cryopreserved following the protocol previously described by our group (41, 43). Semen was diluted to 100 × 10^6^ sperm/mL in TTFM. Before that, the samples were refrigerated by cooling at a rate of −0.25°C/min to 5°C using a water bath in the refrigerated chamber. After 2 h of equilibration at 5°C, the diluted samples were packed into 0.25 mL French straws. Then, using a programmable biofreezer (Kryo 10 Series III; Planes PLC, Sunbury-on-Thames, UK), the straws were frozen by cooling at a rate of −20°C/min to −100°C and finally dropped into liquid nitrogen. Samples were kept in liquid nitrogen containers until being thawed. The straws were thawed in a water bath at 65°C for 5 s.

### 2.3 Sperm motility and kinetic parameters

Sperm motility and kinetic parameters were determined using the CASA system (computer assistant sperm analysis) (Sperm Class Analyzer -SCA- 6.3.0.59; Microptic S.L., Barcelona, Spain). Pooled ejaculates from each male were diluted to 2 × 10^6^ in TES-TRIS-fructose medium supplemented with 1% egg yolk and warmed to 37°C on a warming plate for the samples from each experimental group (breeding season, nonbreeding season, positive control and frozen-thawed samples). Five microliters of the diluted sample was dropped into a Makler counting chamber (10 μm depth; Sefi Medical Instruments, Mumbai, India) and analyzed with the CASA system. The SCA system consisted of an optical phase-contrast Nikon Eclipse microscope (Nikon, Tokyo, Japan) equipped with a Basler acA1300-200uc digital camera (Basler Vision Technologies, Ahrensburg, Germany) and a warmed stage (37°C) and observations were made with a 10x objective with negative phase contrast specifically set for ram spermatozoa (1 μm < particular area < 20 μm^2^). This program was set to capture at 100 frames/second (particles with an area of 20-70 μm^2^). The sperm quality parameters included in our study were the percentage of total motile spermatozoa (TM, %), defined as the percentage of sperm with VCL>15 μm/s; progressive motility (PM, %), defined as the percentage of sperm with VCL>45 μm/s; and certain kinetic parameters: curvilinear velocity (VCL, μm/s) and amplitude of the lateral displacement of the sperm head (ALH, μm). A total of seven sperm samples from each experimental group were analyzed.

### 2.4 Multiparametric flow cytometry analyses

Sperm samples from different experimental groups were analyzed by flow cytometry. Different fluorochromes were combined to evaluate sperm quality: Zombie Violet™ Fixable Viability Kit (excitation 405 nm, emission 423 nm) (Biolegend, San Diego, California, EEUU), used to determine viability associated with membrane integrity; CellEvent™ Caspase-3/7 Green Detection Reagent (excitation 502 nm, emission 530 nm) (Invitrogen, Eugene, Oregon, EEUU), an apoptosis marker; and CellROX™ Deep Red (excitation 644 nm, emission 665 nm) (Invitrogen, Eugene, Oregon, EEUU) and CellROX™ Green (excitation 485 nm, emission 520 nm) (Invitrogen, Eugene, Oregon, EEUU), markers of ROS content. The Zombie Violet™ Fixable Viability Kit is an amine- reactive fluorescent dye that is non-permeant to live cells but permeant to the cells with compromised membranes. Thus, it can be used to assess the live and dead status of mammalian cells by determining two subpopulations: (1) the subpopulation with intact membranes (viable sperm low stained by Zombie Violet™), and (2) the subpopulation showing compromised membranes (dead sperm high stained by Zombie Violet™). Each sample was diluted in PBS (Merck, Madrid, Spain) to obtain a total of 2 × 10^6^ sperm per sample, and the samples were centrifuged (from 0 to 14,100 × g in 12 s and maintained at that speed until 15 s) (MiniSpin Plus, Eppendorf, Hamburg, Germany). The sperm pellet was incubated with 96 μL of Zombie Violet (1:1,000 final dilution), 2 μL of CellEvent Caspase-3/7 (4 μM final concentration) and 2 μL of CRDR (5 μM final concentration) in the dark at room temperature for 30 min. On the other hand, with respect to CRG because it presents the same emission/excitation spectrum as CellEvent™ Caspase-3/7 Green Detection Reagent, it was included with Zombie Violet™ (1:1,000 final dilution) in a separate tube following the same procedure as the other samples (5 uM final concentration of CRG). Different emission/excitation spectrum from each fluorochrome are shown in Supplementary Figure 2. Samples were then washed to stop cell staining, and the pellet was resuspended in 1 mL of PBS. Additionally, following the procedure described above, another study was performed including in the same tube Zombie Violet (1:1,000 final dilution), CRDR (5 μM final concentration) and CRG (5 μM final concentration), analyzing both CellROX™ tests in the same population of viable sperm. This analysis was performed in the best sperm quality group (fresh samples), and in the worst sperm quality group (positive control treated with peroxide samples). Sperm samples were analyzed using a flow cytometer (MACSQuant Analyzer 10, Miltenyi Biotech, Bergisch Gladbach, Germany) equipped with three lasers emitting at 405, 488, and 635 nm (violet, blue and red, respectively) and 10 photomultiplier tubes. Violet fluorescence was detected in V1 (excitation 405 nm, emission 450/50 nm), green fluorescence was detected in B1 (excitation 488 nm, emission 525/50 nm), and red fluorescence was detected in R1 [excitation 635 nm, emission 655–730 nm (655LP + split 730)]. The system was controlled by MACS Quantify software (Miltenyi Biotech, Bergisch Gladbach, Germany), and a total of 40,000 events were recorded for each sample with a flow rate of 200–300 cells/second. Data analysis was carried out by FlowJo v.10.2 (Ashland, Wilmington, DE, USA). In flow cytometry analysis, the population corresponding to the sperm was selected, eliminating the rest of the events (debris). In addition, singlets were selected, eliminating doublets from the analysis. Also, unstained controls were used to determine positive and negative events. Representative cytograms of the assay and gating strategy are shown in Supplementary Figure 3. A total of seven sperm samples from each experimental group were analyzed.

It is important to note that the CRDR probe cannot be fixed with formaldehyde, in contrast to the manufacturer specifications. This fact makes it necessary to read the samples in the flow cytometer at the time of processing. On the other hand, CellROX™ Green probe is fixable with formaldehyde according to the manufacturer's specifications.

### 2.5 CellROX™ Deep Red and Green intracellular location

Two samples from the most differentiated quality experimental groups (fresh sample from the breeding season like highest quality control and positive damage sample treated with 300 μM hydrogen peroxide like lowest quality control) were used to verify the intracellular location of the two CellROX probes. Two hundred microliters (25 × 10^6^ sperm/mL) of each semen sample was washed in PBS and centrifuged (from 0 and 14,100 × g in 12 s and maintained at that speed until 15 s). Sperm cells were incubated with 4 μL of CRG (1 mM) and 4 μL of CRDR (1 mM) with 100 μL of PBS in the dark at room temperature for 30 min following the protocol described by Lanconi et al. (44). After that, another washing step was carried out to stop cell staining, and the sperm cells were resuspended in 50 μL of PBS. Afterward, an aliquot of 5 μL of the stained solution was placed between a slide and coverslip and observed by epifluorescence microscopy (Eclipse Ni-E) (Nikon, Tokyo, Japan) equipped with a Photometrics BSI digital camera (Prime BSI, Photometrics, USA) using a 40 × objective (total magnification: 400 × ). The same field was captured in a light field with a FITC filter (CRG detection) and a Cy5 filter (CRDR detection).

### 2.6 Lipid peroxidation assay

MDA generation was measured with a Lipid Peroxidation Assay kit (Lipid Peroxidation MDA Assay Kit (MAK085), Sigma–Aldrich, Darmstadt, Germany). One million sperm cells were homogenized on ice in 300 μL of MDA lysis buffer containing 3 μL of BHT. Samples were centrifuged at 13,000 × g for 10 min to remove insoluble material. Next, 600 μL of TBA solution was added to form the MDA-TBA adduct in the standard and each sample. Then, the samples were incubated at 95°C for 60 min. After that, the samples were cooled to room temperature in an ice bath for 10 min. Two hundred microliters of each sample reaction mixture were added to a well in duplicate. The fluorescence intensity at 532 nm and 553 nm was measured with a plate reader (Biotek, Gene 5 Microplate Reader, Winooski, VT, USA). A calibration curve was prepared from the MDA standard included on the plate. Finally, the concentration of MDA in the samples was calculated using the equation obtained from linear regression of the standard curve. Seven samples from the same males were analyzed in each of the four experimental groups (twenty-eight samples in total). Analysis was performed in duplicate, and the mean of the two values was taken as the result.

### 2.7 Sperm chromatin structure assay (SCSA)

The SCSA is a technique that quantifies the metachromatic shift from green fluorescence (double-stained DNA) to red (denatured single-stranded DNA). Acridine orange staining was performed following the procedure described by Everson et al. (45). Previously, samples from each experimental group were diluted to 2 million sperm/mL with TNE buffer (10 mM Tris, 150 mM NaCl, 1 mM EDTA, pH 7.4). After that, 0.1 mL of sperm sample was mixed with 0.2 mL of detergent solution composed of 0.1% Triton X-100 in 0.08 N HCl, and 0.15 M NaCl was added to induce partial DNA denaturation. Thirty seconds later, the sperm samples were stained with 0.6 mL of acridine orange solution (6 μg/mL electrophoretically purified acridine orange in citrate-phosphate buffer). Three minutes after staining, the sperm samples were analyzed by flow cytometry. The DNA fragmentation index (DFI) was calculated based on the intensity of red fluorescence divided by the total (red plus green) fluorescence, indicating the amount of denatured sperm DNA relative to the total amount of DNA in each sperm. A cut-off value in DFI at 0.75 was performed to obtain the percentage of sperm with high DFI (hDFI) (20, 23).

### 2.8 Statistical analysis

Prism 8 (GraphPad Software, San Diego, CA, USA) was used to perform statistical analysis. Differences were considered significant when *p* values were < 0.05. Data were analyzed by the Kolmogorov–Smirnov and Levene tests to verify the normality and homogeneity of variances, respectively. Data were evaluated by one-way ANOVA (normally distributed data) or Kruskal–Wallis (nonnormally distributed data). The results are expressed as the mean ± S.E.M. Pearson and Spearman correlation coefficients between seminal parameters were calculated. The reliability of the scoring systems was evaluated by the correlation coefficient (R squared). The number of asterisks (^*^) indicates the level of significance: one asterisk (^*^) indicates *P* < 0.05, two asterisks (^**^) indicate *P* < 0.01, and three asterisks (^***^) indicate *P* < 0.001.

## 3 Results

### 3.1 Multiparametric sperm quality analyses

**Figure 1:**
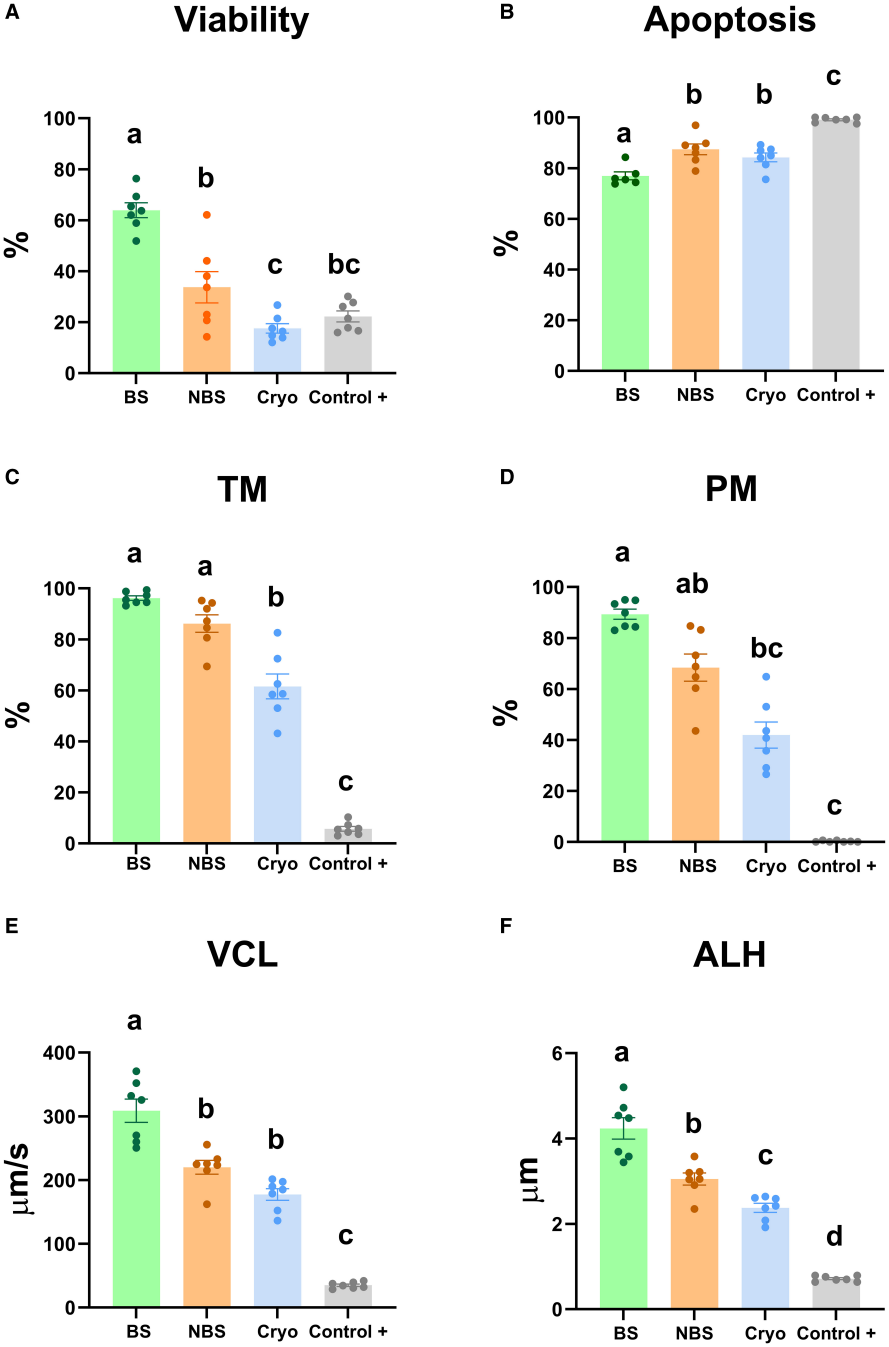
Multiparametric flow cytometry analyses and motility and kinetic parameters of ram sperm from the four experimental groups [fresh in breeding season (BS), fresh in nonbreeding season (NBS), positive control treated with hydrogen peroxide (300 μM) (Control +) and cryopreserved (Cryo)]. **(A)** Viability (%), **(B)** apoptosis (%), **(C)** total motility (TM, %), **(D)** progressive motility (PM, %), **(E)** curvilinear velocity (VCL, μm/s), and **(F)** amplitude of the lateral displacement of the sperm head (ALH, μm). Significant differences (*P* < 0.05) among experimental groups are noted with different lowercase letters (a, b). The same seven males were analyzed in each experimental group.

The viability of the samples from the nonbreeding season, those treated with hydrogen peroxide (positive control) and those frozen-thawed samples decreased significantly (P < 0.05) compared to BS sperm (Figure 1A). However, the viability of the sperm in the NBS and positive control samples were not significantly different (Figure 1A). Similarly, frozen-thawed samples did not show significant differences from the positive control samples (Figure 1A). On the other hand, as expected, apoptosis increased significantly (P < 0.05) in NBS, frozen-thawed and positive control samples in comparison with BS (Figure 1B). Moreover, apoptosis decreased significantly (P < 0.05) in frozen-thawed samples compared to the positive control (Figure 1B). Nevertheless, apoptosis was not significantly different between the NBS and frozen-thawed samples (Figure 1B).

Concerning motility parameters, TM decreased significantly (*P* < 0.05) in the positive control and frozen-thawed samples in comparison with BS (Figure 1C). In contrast, TM did not show a significant difference between the BS and NBS samples (Figure 1C). Moreover, TM decreased significantly (*P* < 0.05) in the positive control compared to the frozen-thawed samples (Figure 1C). PM decreased significantly (*P* < 0.05) in the frozen-thawed samples and positive control compared to the BS samples (Figure 1D). Moreover, PM did not show a significant difference between the positive control and frozen-thawed samples (Figure 1D). Likewise, there was no significant differences between the breeding and nonbreeding season samples (Figure 1D).

VCL decreased significantly (*P* < 0.05) in the NBS, frozen-thawed and positive control samples in comparison with BS (Figure 1E). However, VCL did not show a significant difference between the NBS and frozen-thawed samples (Figure 1E). However, VCL increased significantly (P < 0.05) in frozen-thawed samples compared to positive control samples (Figure 1E). ALH decreased significantly (*P* < 0.05) in the NBS, positive control and frozen-thawed samples in comparison with BS (Figure 1F). Moreover, ALH decreased significantly (P < 0.05) in the positive control and frozen-thawed samples in comparison with NBS (Figure 1F). Nevertheless, ALH decreased significantly (P < 0.05) in the positive control compared to the frozen-thawed samples (Figure 1F).

### 3.2 Redox sperm status

**Figure 2:**
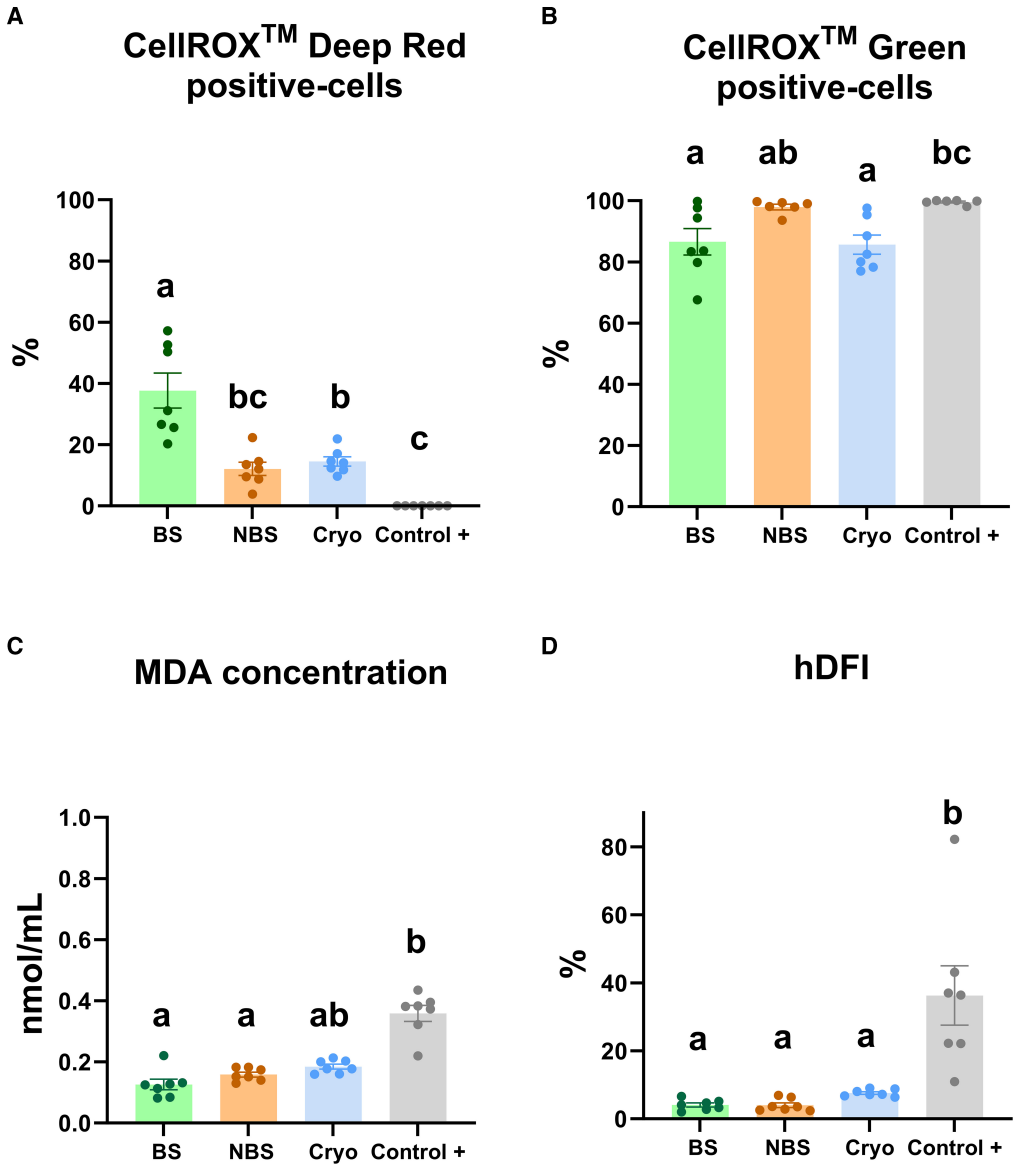
Redox status analyses in ram sperm from the four experimental groups using different assays [fresh in breeding season (BS), fresh in nonbreeding season (NBS), positive control treated with hydrogen peroxide (300 μM) (Control +) and frozen-thawed (Cryo)]. **(A)** CellROX™ Deep Red-positive cells (%), **(B)** CellROX™ Green-positive cells (%), **(C)** MDA generation (nmol/mL), and **(D)** high DNA fragmentation index (hDFI, %). Significant differences (*P* < 0.05) among experimental groups are noted with different lowercase letters (a, b). The same seven males were analyzed in each experimental group.

The CRDR- and CRG-positive sperm populations were characterized by flow cytometry (Figures 2A, B). The number of CRDR-positive cells increased significantly (*P* < 0.05) in BS compared with NBS, positive control and frozen-thawed samples (Figure 2A). However, the NBS samples did not show significant differences between the positive control and frozen-thawed samples in CRDR-positive cells (Figure 2A). In contrast, the number of CRDR-positive cells decreased significantly (P < 0.05) in the positive control samples in comparison with the frozen-thawed samples (Figure 2A). Nevertheless, the number of CRG-positive cells increased significantly (*P* < 0.05) in the positive control compared to the BS and frozen-thawed samples (Figure 2B). In contrast, BS did not show significant differences from the NBS and frozen-thawed samples (Figure 2B). Both MDA generation and the high DNA fragmentation index increased significantly (*P* < 0.05) in the positive control samples in comparison with the fresh samples from breeding and nonbreeding seasons (Figures 2C, D). Likewise, the MDA concentration and high DNA fragmentation index did not show significant differences from the BS and NBS fresh samples (Figures 2C, D). Moreover, the MDA concentration was not significantly different between the frozen-thawed and positive control samples (Figure 2C), while the high DNA fragmentation index increased significantly (*P* < 0.05) in the positive control samples in comparison with the frozen-thawed samples (Figure 2D).

**Figure 3:**
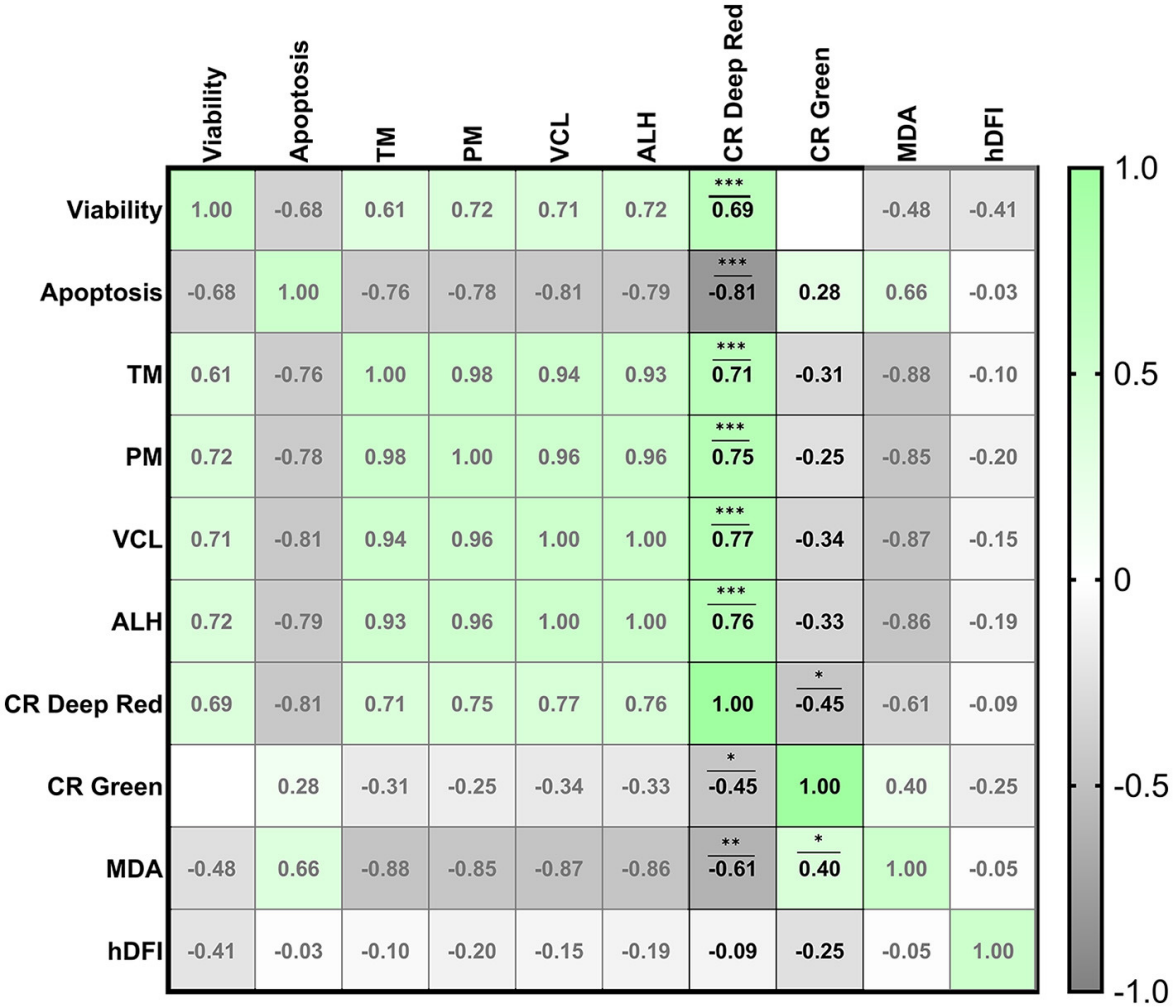
Correlation matrix of all sperm quality markers evaluated highlighting the correlations between the CellROX™ Deep Red and CellROX™ Green fluorescent probes. The four experimental groups [fresh samples from breeding and nonbreeding seasons, frozen-thawed and positive control treated with hydrogen peroxide (300 μM)] were included to construct the correlation matrix. Viability (Zombie-negative cells), apoptosis (caspase-3/7-positive cells), TM (total motility), PM (progressive motility), VCL (curvilinear velocity), ALH (amplitude of the lateral displacement of the sperm head), CR Deep Red (CellROX™ Deep Red-positive cells), CR Green (CellROX™ Green-positive cells), MDA (malondialdehyde generation), and hDFI (high DNA fragmentation index). The R squared value between two parameters is noted in each cell. Blue indicates positive correlations, and red indicates negative correlations. The color intensity represents the strength of the correlation between two sperm quality parameters. A total of seven males were analyzed (the same number of samples is required for this analysis). Asterisks show significant correlations among sperm quality parameters. The number of asterisks (*) indicates the level of significance: (*) indicates *P* < 0.05, two asterisks (**) indicate *P* < 0.01, and three asterisks (***) indicate *P* < 0.001.

Different correlations among quality parameters were found when we analyzed sperm viability, apoptosis, MDA concentration and motility and certain kinetic parameters when comparing with CRDR and CRG (Figure 3). Some correlations were also found among CRDR- and CRG-positive cells in terms of the other sperm quality parameters. On the one hand, CRDR-positive cells presented significant positive correlations with viability (R^2^=0.69), motility and kinetic parameters (TM (R^2^=0.71), PM (R^2^= 0.75), VCL (R^2^= 0.77), ALH (R^2^= 0.76)) (*P* < 0.001) (Figure 3). On the other hand, CRDR-positive cells presented significant negative correlations with apoptosis (R^2^ = −0.81) (*P* < 0.001) and MDA concentration (R^2^ = −0.61) (*P* < 0.01) (Figure 3). CRG-positive cells presented a significant positive correlation with MDA concentration (R^2^= 0.40) (*P* < 0.05) showing an opposite correlation pattern with CRDR probe (Figure 3).

Moreover, the correlations of the two CellROX™ probes with each other have been studied. A significant negative correlation was found between CRDR- and CRG-positive cells (R^2^ = −0.45) (*P* < 0.05) (Figure 3).

Additionally, with respect to CellROX tests performed on viable sperm population, viable CRDR-positive cells were significantly (P < 0.001) increased in fresh compared to the positive control samples (Figure 5A). In contrast, viable CRG-positive sperm were significantly increased in the positive control compared to fresh samples (*P* < 0.05) (Figure 5B).

### 3.3 CellROX™ localization in sperm samples

**Figure 4:**
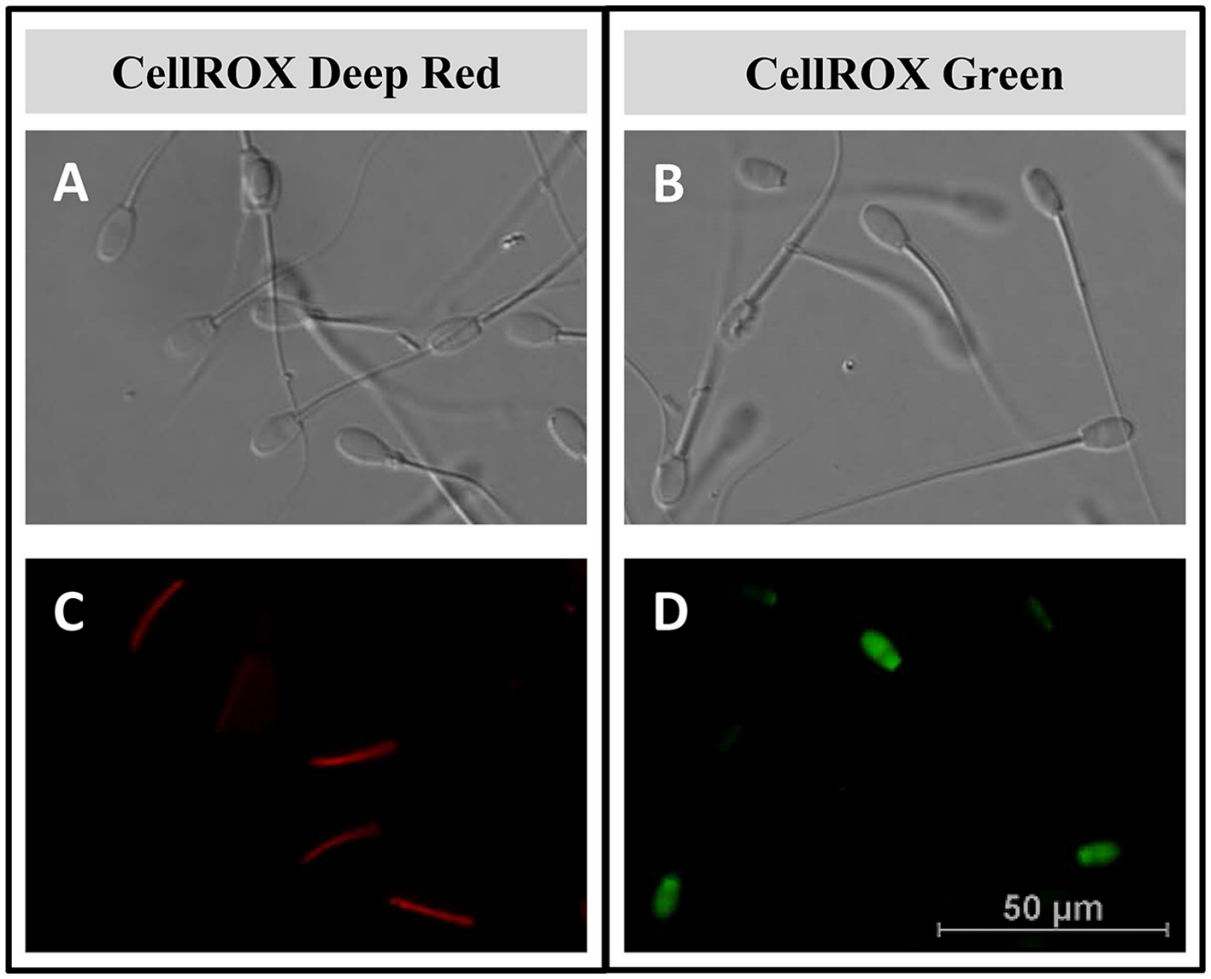
Intracellular reactive oxygen species localization by confocal microscopy in ram sperm samples. **(A, B)** Light field image of ram sperm samples. **(C)** Middle piece of the sperm stained red with the CellROX™ Deep Red fluorescent probe. **(D)** Sperm nucleus stained green with the CellROX™ Green fluorescent probe.

**Figure 5:**
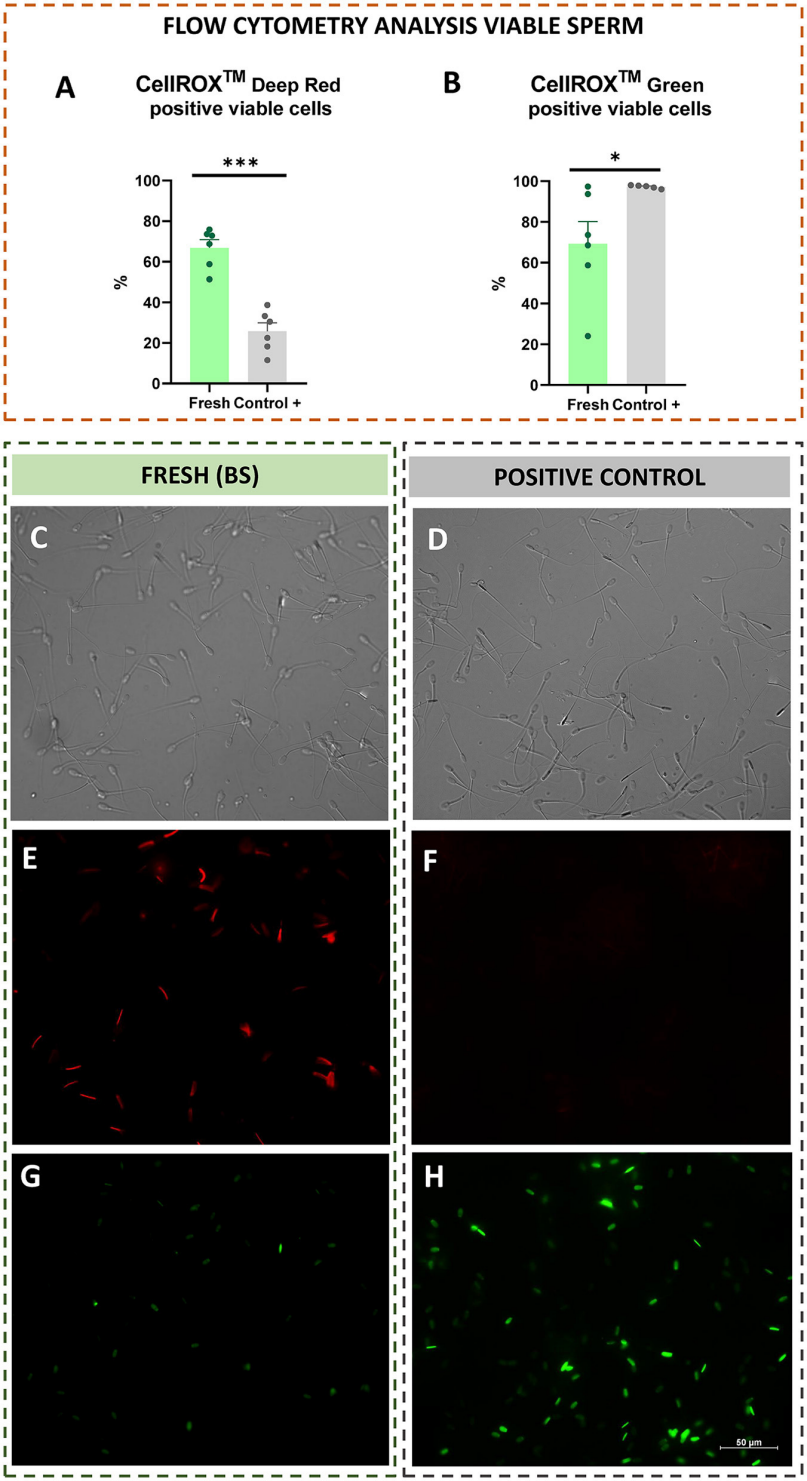
Flow cytometry analysis from viable sperm (CellROX™ Deep Red and Green), and intracellular reactive oxygen species localization by confocal microscopy in fresh (breeding season) and positive control [treated with hydrogen peroxide (300 μM)] ram sperm samples. **(A)** CellROX™ Deep Red positive viable sperm. **(B)** CellROX™ Green positive viable sperm. **(C)** Light field image of the BS sample. **(D)** Light field image of the positive control sample. **(E)** CellROX™ Deep Red fluorescent probe in the BS sample (middle piece stained red). **(F)** CellROX™ Deep Red fluorescent probe in the positive control sample (middle piece not stained). **(G)** CellROX™ Green fluorescent probe in the BS sample (nucleus not stained). **(H)** CellROX™ Green fluorescent probe in the positive control sample (nucleus stained green). The number of asterisks (*) indicates the level of significance: one asterisk (*) indicates *P* < 0.05, and three asterisks (***) indicate *P* < 0.001.

The intracellular location of CellROX™ was determined by confocal microscopy. During the breeding season, the middle piece of the sperm cells was stained red by CRDR (Figures 4A, C, 5C, E). However, sperm in the positive control group did not show their middle piece stained red after incubation with CRDR (Figure 5F). Concerning CRG, sperm cells from the positive control sample showed green nuclear staining (Figures 4B, D, 5D, H), while sperm cells from the BS sample did not show nuclear staining (Figure 5G). Furthermore, with respect to the number of stained sperms, the highest number of CRDR-positive cells was registered in the best quality sample (breeding season) (Figures 5C, E), while in the case of CRG, the highest number of positively stained cells was detected in the positive damage control group (Figures 5D, H).

## 4 Discussion

Redox balance is essential for living systems, including sperm cells (46). The balance between electron loss (oxidation) and electron gain (reduction) is crucial for adequate cellular functionality (11, 12, 47). Therefore, oxidative stress occurs when ROS generation exceeds the protective capacity of the antioxidant mechanisms (11, 12, 46). This can be due to a lack of antioxidant protection or the overproduction of ROS (11, 12, 46). Excessive ROS production may be cytotoxic to sperm, causing a decrease in sperm quality (4, 48). Furthermore, it should be noted that ROS are necessary for the normal physiological functions of sperm and are involved in important signal transduction pathways (40). Moreover, ROS are required for sperm chromatin compaction, providing protection against oxidative DNA damage (49).

Recently, some fluorescent probes, such as CellROX™ Deep Red and CellROX™ Green, have been described as markers of sperm damage, as they can evaluate the presence of specific ROS in different mammalian species (35, 38, 44). CRDR has been considered an ROS marker in ram (35, 50), bull (38, 51) and stallion sperm (44, 52–54). However, contradictory results have been published concerning their correlation with sperm quality in rams (42, 55, 56) and stallions (57, 58). These results could suggest that there may be a species-specific effect, which we have decided to study in depth.

New markers based on redox status, including deep characterization of ROS probes, could be very useful to analyze sperm quality to optimize ram sperm preservation protocols (cooling and freezing). For this purpose, four experimental groups with different initial sperm qualities were established. These experimental groups included fresh (BS and NBS), frozen-thawed and positive control samples (as a damage control). Afterward, multiparametric analyses of the sperm, including viability, apoptosis, lipid peroxidation, motility and kinetic parameters (VCL and ALH), were applied to corroborate the differences in quality of the previously established experimental groups. As expected, after analyzing the quality of the semen from the different experimental groups, two were of high quality with low levels of oxidative stress (fresh samples in the BS and NBS groups). In addition, two groups with low sperm quality and a high level of oxidative stress (thawed samples provided in the NBS and positive damage control samples) were obtained. However, Mendoza et al. (59) previously described increases in apoptotic markers, such as phosphatidylserine translocation, mitochondrial membrane potential or caspase activation, in samples from the nonbreeding season compared with those from the breeding season. Apoptosis appears to be triggered by oxidative stress, which leads to the activation of ROS generation (60). In addition, we chose the experimental groups with more damage (frozen-thawed and positive control) because they were considered the most suitable to experience oxidative stress and therefore an imbalance in ROS. On the one hand, frozen-thawed samples are subject to oxidative stress due to the drastic temperature changes during the cryopreservation process or the high dilution rates to which they are subjected (61, 62). As previously demonstrated, caspases 3–7 are activated, triggering apoptosis as a consequence of drastic temperature change (cryopreservation and thawing process) as a result of the generated ice crystals, which could result in damage to the sperm (42, 61, 63). On the other hand, the positive control samples were incubated with hydrogen peroxide as described by Soliman et al. (16). Oxidative stress induced by hydrogen peroxide causes a drastic decrease in sperm viability, as several authors have previously demonstrated (16, 64). Furthermore, Pujianto et al. (65) demonstrated that a high concentration of hydrogen peroxide is responsible for oxidative stress as a consequence of excessive ROS production in sperm that causes a decrease in sperm quality, including an increase in cell apoptosis. As expected, our results revealed that the fresh BS samples were of the highest quality among the experimental groups. The BS samples showed the highest viability and motility and kinetic parameters (TM, PM, VCL, ALH) and the lowest level of apoptosis (Figures 1A–F) compared to the group in which oxidative stress was induced (positive control samples). Some authors previously described this detrimental effect in oxidative stress-induced samples (16). With respect to the fresh samples inside and outside the reproductive season, while apoptosis significantly increased in the NBS group compared to the BS group, the viability and kinetic parameters increased significantly in the BS compared to the NBS samples (Figures 1A–F). This was reported previously by Kafi et al., who described lower viability in winter than in summer in Karakul rams located in Shiraz (Iran) (66). Moreover, we included cryopreserved sperm samples as a group with intermediate quality that gave values between the fresh and peroxide-treated samples for most of the analyzed parameters. As previously described by several authors and according to our results, cryopreservation induces detrimental effects to sperm quality parameters, including motility and viability (16, 42). To improve sperm quality analyses and confirm the initial differences among the experimental groups in terms of ROS production, the MDA concentration was used as a traditional marker for the assessment of lipid peroxidation and therefore oxidative stress (16). In our results, contrary to previous studies in rams, lipid peroxidation increased significantly in samples assessed after incubation with hydrogen peroxide for 24 h (positive damage control) compared to fresh samples (Figure 2C) (16). This may be because, in contrast to our study, Peris et al. (16) used synthetic oviduct fluid (SOF) as a diluent for both the fresh and peroxide-treated samples. However, we did not observe any differences in lipid peroxidation between the fresh and thawed samples, which is in agreement with the results of Soliman et al. (16). In accordance with previous studies in rams, a similar trend was observed for hDFI, which increased significantly in the positive control samples compared to the fresh samples (16). However, the absence of a difference between the fresh and thawed samples could be explained by the low DNA fragmentation index in rams compared to the other species (67). These results indicate that more accurate ROS tests should be performed to discriminate between fresh samples and cryopreserved samples in rams.

Consequently, after analyzing the differences in sperm quality among the experimental groups, we performed a specific study of two commercial ROS detection probes (CRG and CRDR) for ram sperm quality evaluation.

Our results revealed that CRDR and CRG gave opposite labeling patterns. The number of CRDR-positive cells was higher in fresh BS samples than in frozen-thawed and positive control samples (Figure 2A). Because of this, some significant positive correlations were found between CRDR-positive cells and positive markers of sperm quality, including motility and viability (Figure 3). Furthermore, attending to the viable sperm population, a higher expression of CRDR-positive cells was observed in fresh samples compared to peroxide-treated samples (Figure 5A). In accordance with our results, some authors have previously hypothesized that CRDR is a positive quality marker in stallion sperm (57, 58). Davila et al. (57) have previously included the CRDR probe in their studies, where they demonstrated that it measures superoxide production. Their studies showed that an increased concentration of superoxide anion in mitochondrial sperm was associated with intense mitochondrial activity. Moreover, these authors also observed positive correlations with motility and membrane integrity in stallion sperm. This could be explained by the fact that the blocking electron transfer caused a disruption in production of superoxide anion, which is the target molecule of CRDR. In addition, other studies have shown that 1% to 2% of superoxide anion (O_2_) used in the electron transport chain is not completely reduced, generating the O_2_ (68). These observations are in accordance with our results, where sperm samples affected by cryopreservation or peroxide treatment suffered a decrease in superoxide anion production, triggering a decrease in CRDR labeling. For this reason, considering that the CRDR probe mainly detects this anion, an increase in the O_2_ is associated with high mitochondrial activity rather than oxidative stress. On the other hand, CRG has been described as a ROS marker in humans (69) and bulls (36, 70). Unlike CRDR, the number of CRG-positive cells was higher in the frozen-thawed and positive control samples than in the breeding season samples (Figure 2B). Moreover, some significant positive correlations were found between CRG-positive cells and negative sperm quality markers, such as apoptosis and MDA (Figure 3). Likewise, attending to the viable sperm population, a higher expression of CRG-positive cells was also observed in peroxide-treated samples compared to fresh samples (Figure 5B). Our results were in accordance with previous works that demonstrated an increase in the percentage of CRG-positive cells after exposure to an oxidative environment (36). Moreover, de Castro et al. demonstrated that this probe was a very sensitive marker in bull sperm (36). They observed a dose-dependent effect, and CRG fluorescence intensity increased with increasing concentrations of hydrogen peroxide (36). However, the CRG probe has not yet been studied in ram sperm. According to the manufacturer's instructions, and as described by Riley et al. (69) in their study, the CRG probe has weak basal fluorescence that increases as the sample becomes oxidized (Supplementary Figure 4). Based on our results, it is necessary to include all the available techniques for CellROX characterization, including intracellular location, which is a crucial factor (40). In addition to the opposite labeling pattern, our studies revealed different intracellular locations of the CellROX fluorescent probes as studied by confocal microscopy. CRDR fluorescence was located in the middle piece of the sperm (Figure 4C) as previously published by Rodrigues et al. (35). These authors found that only sperm subjected to oxidative stress showed red fluorescence. Contrarily, our study only detected red fluorescence in fresh samples (35). This inconsistency may be due to the solvent used for the oxidative stress induction agent since Rodrigues et al. (35) used TALP medium, which has antioxidants in its composition and could had had some interaction during samples incubation.

While in our study we used hydrogen peroxide, Rodrigues et al. used ferrous sulfate and sodium ascorbate (35). This could mean that the medium used by Rodrigues et al. did not induce oxidative stress as effectively, since in our study CRDR fluorescence correlated with the most parameters of seminal quality and oxidative stress. This could be due to the antioxidant effect of Tyrode's Albumin Lactate Pyruvate (TALP) medium that could neutralize the effect of the agents used by Rodrigues et al. to induce oxidative stress. The components of the TALP medium contain pyruvate and lactate, components with an antioxidant effect and promoting mitochondrial functionality as previously described (71, 72). In addition, mitochondria are located in the midpiece of the sperm, which is the location of the highest metabolic activity in viable sperm (73). Therefore, the localization of CRDR fluorescence in the midpiece of the fresh samples could be a useful indication of proper sperm functionality. On the other hand, we observed that CRG fluorescence was located in the sperm nucleus in positive control samples (Figures 4D, 5H). In accordance with our results, CRG fluorescence was observed in the nuclei of bull sperm subjected to hydrogen peroxide treatment (36).

These results demonstrated that CRG and CRDR presented an opposite labeling pattern that was corroborated by fluorescence microscopy that showed different localization of the probes. While CRDR fluorescence was observed in the middle piece of the sperm from the fresh samples with superoxide anion production (intense mitochondrial activity sperms), CRG was found in the nuclei of the sperm in the positive control samples and was associated with DNA damage. These results could be explained by the fact that CRG is a DNA dye, and upon oxidation, it binds to DNA; thus, its signal is localized primarily in the nucleus and mitochondria. This fact was previously demonstrated by in a ROS localization study in *Solea senegalensis* sperm (40). In contrast, the signals from CRDR were localized in the cytoplasm. These results could hypothesize that in sperm, the localization of ROS is a crucial factor in sperm quality determination, as in previous studies performed in fish (40). Furthermore, we can conclude from our results that CellROX probes could be more discriminatory tests for the assessment of oxidative stress than lipid peroxidation measurements or DNA fragmentation. This is because, unlike the evaluation of lipid peroxidation, the use of CRDR and CRG allowed us to establish differences between the two experimental groups (frozen-thawed and peroxide-treated samples). Furthermore, we could consider that CRDR is a more accuracy test compared to CRG according to the highest correlations observed with the semen quality parameters studied (Figure 3). In addition, differences in the significance of both tests were also observed in viable sperm: CRDR (*P* < 0.05) shows a high level of significant differences between the two extreme groups compared to CRG (*P* < 0.001). This work allowed us to characterize CRG and CRDR with fluorescence assays for use as innovative and accurate tools in seminal assessment by focusing on the redox status of ram sperm cells. In this study, we observed the relationship between intracellular ROS location and the effect of ROS on sperm cells. CRDR fluorescence was located in the sperm mitochondrial region (middle piece) in the fresh sperm samples, which could be associated with high mitochondrial activity according to viability and motility analyses performed in this work. On the other hand, CRG fluorescence was mostly located in the cell nuclei in the positive control and frozen-thawed samples, which could be related with sperm damage, due to its positive correlation with detrimental sperm quality parameters such as MDA concentration.

## Data availability statement

The original contributions presented in the study are included in the article/Supplementary material, further inquiries can be directed to the corresponding author.

## Ethics statement

The animal study was approved by Animal Care and Use Committee of the University of León (Spain) ÉTICA-ULE-050-2022. The study was conducted in accordance with the local legislation and institutional requirements.

## Author contributions

CP-M: Conceptualization, Data curation, Formal analysis, Investigation, Methodology, Visualization, Writing – original draft. LA-L: Conceptualization, Data curation, Funding acquisition, Methodology, Project administration, Resources, Visualization, Writing – review & editing. MA: Conceptualization, Project administration, Resources, Writing – review & editing. MN-M: Conceptualization, Investigation, Methodology, Formal analysis, Visualization, Writing – review & editing. RM-G: Conceptualization, Investigation, Methodology, Formal analysis, Visualization, Writing – review & editing. CS-Ú: Conceptualization, Resources, Writing – review & editing. PP: Conceptualization, Resources, Writing – review & editing. LA: Conceptualization, Funding acquisition, Project administration, Resources, Writing – review & editing. MR: Conceptualization, Methodology, Resources, Supervision, Validation, Writing – review & editing.

## Supplementary material

Supplementary Figure 1 Experimental groups used in the experiment: fresh samples (breeding season (BS) and non-breeding season (NBS)), frozen-thawed provided from NBS samples and positive control group provided from BS samples (oxidative stress induction by hydrogen peroxide).

Supplementary Figure 2 Emission spectra of the fluorochromes provided by the commercial manufacturer: Zombie Violet™ Fixable Viability Kit, CellEvent™ Caspase-3/7 Green Detection Reagent, CellROX™ Deep Red, and CellROX™ Green.

Supplementary Figure 3 Representative cytograms of the assays reported in the present study. **(A)** Dot plot showing the region gated corresponds to sperm. **(B)** Events gated in **(A)** are now plotted against SSC-H and SSC-A for select single cells, and this region was further used to set the remaining populations of interest. **(C)** Unstained control for Zombie Violet™, Caspase-3/7 Green, CellROX™ Deep Red, and CellROX™ Green. **(D)** FlowJo analyses for viability (Zombie Violet™). **(E)** FlowJo analyses for apoptosis (Caspase-3/7 Green). **(F)** FlowJo analyses for mitochondrial functionality (CellROX™ Deep Red). **(G)** FlowJo analyses for ROS content (CellROX™ Green).

Supplementary Figure 4 FlowJo analyses of CellROX™ Green by flow cytometry. **(A)** Sperm sample without the fluorochrome CellROX Green. **(B)** Fresh sample from the breeding season. **(C)** Sample from the positive control group (hydrogen peroxide-treated).
