# Supplementary figures and images for: The characterization of CellROX™ probes could be a crucial factor in ram sperm quality assessment
Source: Front Vet Sci. 2024 Feb 27;11:1342808. doi: 10.3389/fvets.2024.1342808 (PMC10927726; doi:10.3389/fvets.2024.1342808)

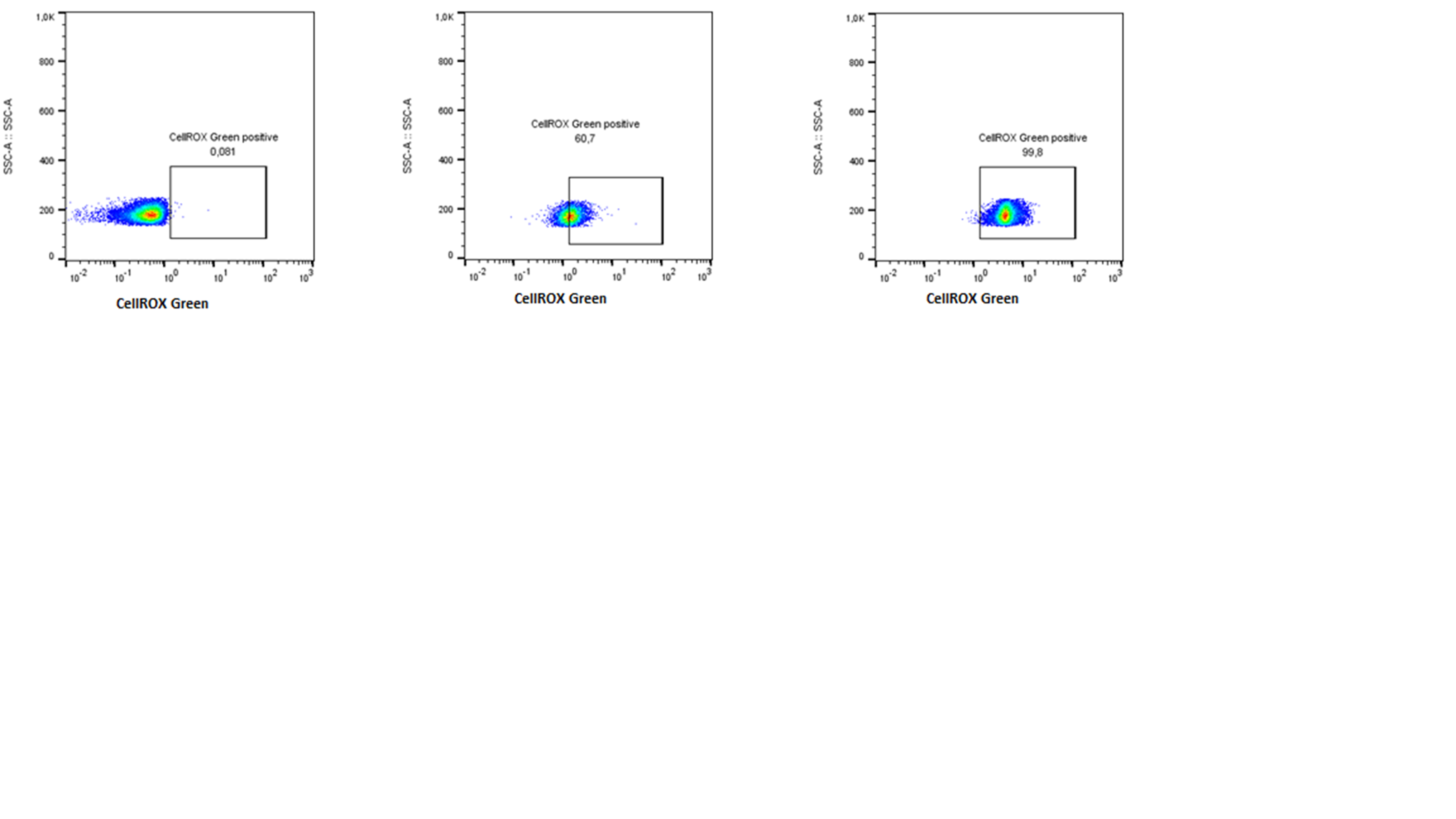

Supplement: Supplementary Figure 4 — FlowJo analyses of CellROX™ Green by flow cytometry. (A) Sperm sample without the fluorochrome CellROX Green. (B) Fresh sample from the breeding season. (C) Sample from the positive control group (hydrogen peroxide-treated). [file Image_4.TIF]
